# Season, size, and sex: factors influencing monogenean prevalence and intensity on *Gambusia affinis* in New Zealand

**DOI:** 10.1007/s00436-024-08241-x

**Published:** 2024-05-31

**Authors:** Ethan D. Renner, Ian C. Duggan

**Affiliations:** Te Aka Mātuatua - School of Science, The University of Waikato, Hamilton, New Zealand

**Keywords:** Parasites, Seasonality, Platyhelminthes, Mosquitofish, *Salsuginus*

## Abstract

A number of studies have been conducted on monogenean seasonality, though primarily in continental regions with wide annual temperatures ranges. We investigated seasonal changes in the prevalence and intensity of *Salsuginus seculus* infesting sexually dimorphic western mosquitofish (*Gambusia affinis*) in New Zealand. This represents the first examination of seasonality for this species globally, and the first seasonal assessment of any monogenean population in New Zealand, a temperate country with a mild oceanic climate. Prevalence and intensity of *S. seculus* with respect to fish size and sex was also examined. Prevalence of *S. seculus* changed temporally, peaking in summer, and was strongly positively correlated with algal concentrations. This relationship may be associated with increasing food levels, leading to an increase in fish courting and mating, resulting in high numbers and close physical associations of *G. affinis* individuals, facilitating transmission of the monogeneans. Thus, biotic factors may be important in determining temporal changes in *S. seculus* prevalence in New Zealand. Female *G. affinis* had a significantly higher prevalence and mean intensity of *S. seculus* than males. Longer fish had a higher mean intensity and prevalence of *S. seculus*. Female *G. affinis* likely host disproportionately more monogeneans as they are larger than males. Alternatively, females may have a compromised immune response during reproductive periods. Overall, seasonal change was observed in *S. seculus* prevalence and intensity under New Zealand’s mild climatic conditions, and the larger female *G. affinis* in this dimorphic species supported a greater prevalence and intensity of infestation than males.

## Introduction

Monogenea are a polyphyletic group of ectoparasitic Platyhelminthes, comprised of the classes Monopisthocotylea and Polypisthocotylea (Brabec et al. 2023). These parasites most commonly infest fish, attaching themselves to surfaces such as the gills, body, and fins, primarily by means of an organ called the haptor (Whittington and Chisholm 2008). Such infestations may occasionally, though not typically, lead to disease (Whittington and Chisholm 2008). Unlike other platyhelminth parasites, such as those in the Digenea, monogeneans have a single-host life cycle and any single monogenean species typically infests only a single host species (Poulin 1992). With the exception of the viviparous members of the Gyrodactylidae family, which produce fully grown young able to transmit directly to new hosts via brief contact between fish (Tepox-Vivar et al. 2022), monogeneans release eggs directly into the water column that hatch into ciliated larval oncomiracidia. These oncomiracidia then swim to find and attach to a prospective host where they mature into adult parasites (Chubb 1977; Whittington and Chisholm 2008).

A number of studies exist on seasonal changes in monogenean populations globally (e.g., Chubb 1977; Valtonen et al. 1990; Mo, 1992; Gutierrez and Martorelli 1994; Ozer and Erdem 1999; Dávidová et al. 2005; Aydogdu 2006; Blažek et al. 2008; Madanire-Moyo et al. 2011; Poulin 2020; Li et al. 2022; Mo et al. 2023). Seasonal changes in parasitological indices such as prevalence and intensity have most commonly been associated with seasonal variability in water temperature. Often, freshwater monogenean species increase in abundance when water is warmer during the summer months in temperate regions (Ozer and Erdem 1999; Aydogdu 2006; Poulin 2020). In the tropics, however, where temperature is more stable throughout the year, freshwater monogenean infestations do not appear to change significantly among seasons (Poulin 2020). Despite this general trend, some monogenean species in temperate regions show greater prevalence and abundance in colder months (Valtonen et al. 1990; Li et al. 2022), while others show multiple instances of high prevalence and intensity throughout the year (Chubb 1977; Li et al. 2022). It is possible that variability in other environmental factors, such as pH and the concentration of dissolved oxygen, may also be relevant, as unlike endoparasites, ectoparasites are exposed to external conditions, which may influence temporal parasite population changes alongside temperature.

Temperate regions where studies on seasonal changes in monogenean populations have been undertaken include the Czech Republic (Dávidová et al. 2005; Blažek et al. 2008), England (Chappell 1969), Finland (Valtonen et al. 1990), Poland (Prost 1963; Wierzbicka 1974), South Africa (Madanire-Moyo et al. 2011), Turkey (Ozer and Erdem 1999; Aydogdu 2006), and the USA (Crane and Mizelle 1968; Rawson and Rogers 1972). With the exception of South Africa, these are all in northern temperate continental areas or are close to such continents, and thus experience wide ranges in temperature through the year (Duckson 1987). In contrast, New Zealand is an isolated set of islands and does not experience the same extremes in temperatures as in continental climates (Green et al. 1987). New Zealand is described as having a mild oceanic climate. That is, it is generally wet, windy, and most notably experiences a relatively narrow range in annual temperatures, with cooler summer temperatures and comparatively warmer winters than northern temperate localities of similar latitude (Green et al. 1987). The unique seasonal and climatic characteristics of New Zealand and its lakes makes it an ideal location in which to examine whether monogenean populations will continue to experience large population changes without large annual changes in temperature.

The western mosquitofish, *Gambusia affinis*, native to the south-eastern USA and Mexico, has established non-native populations in a number of locations globally (Pyke 2008). *Gambusia* live in stagnant or slow moving waters with wide temperature ranges (Rivas 1963) and can tolerate temperatures from 0 to 39 °C (Cherry et al. 1976), though they typically prefer water temperatures from 31 to 35 °C (Pyke 2005), and will undertake diurnal migrations seeking areas of preferred temperature (Maglio and Rosen 1969; Winkler 1979). Examples of annual air temperature ranges in the native distribution of *G. affinis* between 1991 and 2020 include an average annual minimum of − 4.4 °C to an average annual maximum of 31.7 °C in southern Illinois, USA (NOAA 2023). In Texas, USA, the average annual minimum temperature from 1991 to 2020 was 3.6 °C and the average annual maximum was 34.2 °C (NOAA 2023). Such a wide tolerance to temperature has allowed *G. affinis* to successfully establish populations across much of the world (Pyke 2008). In its native range, the breeding season extends from mid-spring to mid-autumn (Pyke 2005). During this time, males and females gather together and several males will cluster around a female. The largest male will chase away smaller competitors and engage in aggressive pursuit of the female. Males use their modified anal fin, known as a gonopodium, to internally fertilize the oocytes of females. These are retained by the female until she gives birth to live young. The gestation period may range from 20 to 25 days but may be as short as 15 days in warmer waters. When the female does give birth, it may be to a clutch of 100 young (Pyke 2005). *Gambusia affinis* plays host to several monogenean species, including *Salsuginus seculus*, *Salsuginus bermudae* Rand and Wiles, 1987, and *Gyrodactylus gambusiae*. The monogeneans of *Gambusia affinis* have been the subject of various ecological studies (Mizelle and Arcadi 1945; Rogers and Welborn Jr 1965; Hanek and Fernando 1972; Nitta and Nagasawa 2014; McAllister et al. 2015; Vasquez 2016; Carpenter and Herrmann 2020). However, the seasonal changes of prevalence and intensity of specific species have not been previously studied.

Prevalence and intensity of monogenean infestations may change with season, but the likelihood and intensity of infestations may also vary from host to host within a single species based on characteristics of the host individual. For example, it has been suggested that larger individual fish within a population may be infested more frequently and may harbor greater numbers of ectoparasites, though evidence for this is variable (Kuris et al. 1980; Rohde et al. 1995; Poulin 2000; Rubio-Godoy 2008; Madanire-Moyo et al. 2011). Large fish present a greater surface area onto which monogeneans may attach and therefore, offer a more favorable habitat patch. Further, larger fish have greater gill area as a function of body mass (Muir 1969; Morand 2000). Larger fish will also typically be older, and as such have had more time to acquire parasites. In the case of *G. affinis*, individuals reach a maximum length of 60 mm (McDowall 1990) and usually live no longer than 15 months (Pyke 2008). As such, individuals that have reached a length of 50 mm or more are likely to be relatively old. On this basis, *G. affinis* individuals that are large and old would be expected to see greater parasite prevalence and intensity. However, patterns in host age and parasite infection are not universal and younger individuals may possess more parasites than their older counterparts (Wunderlich et al. 2022). In parasitic interactions, the immune system of the host is an important factor, and this is not an exception for monogeneans infesting either the skin or gills (Buchmann 1999; Buchmann and Lindenstrøm 2002). Testosterone is often considered to suppress immune function (Foo et al. 2016; Roved et al. 2017), and as male teleosts have higher levels of testosterone than females (Borg 1994), it is possible that more males will be infested with monogeneans and have higher intensities of infestation. This has been observed in *Salmo trutta*, where sexually mature males had a higher prevalence and intensity of ectoparasites (Pickering and Christie 1980). Nevertheless, sex differences in fish parasitism have been investigated in other instances, where no significant difference between sexes have been found (Poulin 1996; Barse 1998; Madanire-Moyo et al. 2011; Calhoun et al. 2018; Carpenter and Herrmann 2020). As *G. affinis* shows marked sexual dimorphism, with mature males being overall smaller than females and possessing a distinct gonopodium (McDowall 1990), it presents an ideal model species on which to test the hypothesis that males suffer from greater infestation by monogeneans.

*Salsuginus seculus* (Family Ancyrocephalidae) and *Gyrodactylus gambusiae* (Gyrodactylidae) have recently been reported infesting *Gambusia affinis* in New Zealand (Renner and Duggan 2023), and this study aimed to investigate the ecology of these species. The temporal changes in monogenean populations on *G. affinis* in ponds on the Hamilton campus of the University of Waikato were studied by recording their prevalence and mean intensity on a monthly basis alongside environmental variables. In doing so, we tested whether monogenean populations would vary significantly over the course of a year in a mild climate and, if so, what factors were responsible for this change. Further, to determine whether the size or sex of *G. affinis* hosts has any effect on the prevalence or intensity of *S. seculus*, these host characteristics were tested in relation to the aforementioned parasitological indices. That is, we tested the hypothesis that larger fish make better habitat patches for parasites, thereby leading to greater prevalence and intensity, and that male fish have lower immunocompetence, and as a result will have greater prevalence and intensity of *S. seculus*.

## Materials and methods

### Field and laboratory analyses

**Fig. 1:**
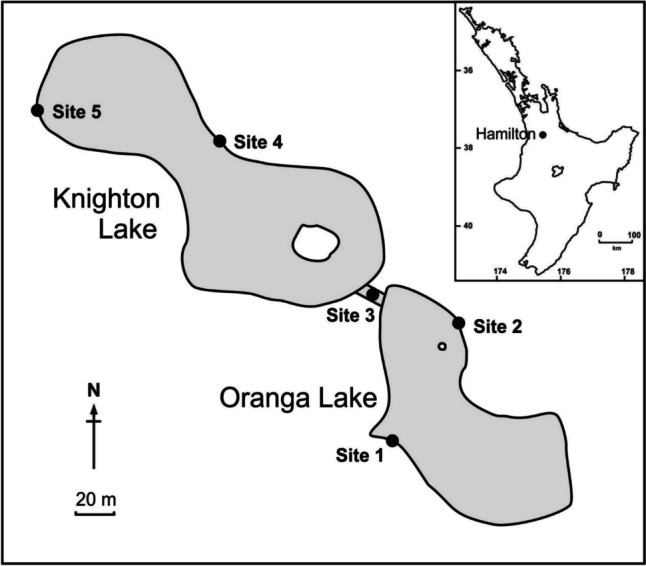
Locations of the five sampling sites in Knighton and Oranga Lake at the University of Waikato, Hamilton campus

From April 2022 to March 2023, sampling for *G. affinis* was carried out in Knighton and Oranga lakes (37° 47′ 08.7″ S, 175° 18′ 53.3″ E and 37° 47′ 12.4″ S, 175° 18′ 57.2″ E, respectively), two connected ponds on the Hamilton campus of the University of Waikato, New Zealand. Oranga Lake has a surface area of 0.69 ha and a maximum depth of 0.6 m, and Knighton Lake 1.01 ha and 0.6 m (Hicks and Bryant 2002). Minnow traps were set at five sites around the shoreline of the ponds on a monthly basis, with numbers supplemented with pole netting; two sites were in Oranga Lake, one in the connecting stream, and two in Knighton Lake (Fig. 1). These were set near vegetation where possible, though little marginal vegetation exists around most of the shoreline of these ponds. In April and May 2022, traps were left at each site for two to four hours. However, due to decreasing numbers of fish caught throughout the remainder of the study, subsequent traps were left for approximately 24 h. It was intended that at least 50 individuals from each of the two lakes and the stream would be caught (i.e., a total of 150 per month). On a few occasions, fish were scarce, and thus fewer than 50 fish were examined for monogeneans from some sites. For example, fish could not be collected for Oranga Lake and Knighton Lake in July 2022 or from the connecting stream in August 2022, possibly due to low fish abundance during the winter. The lowest number of fish examined on any date was 50, in July 2022.

Environmental factors were measured when fish were collected, usually around mid-day. Temperature (°C) and dissolved oxygen concentration (mg L^−1^) were measured 15 cm below the surface at each of the five sites using a YSI meter. At the water’s surface, pH was measured using a pHTestr handheld pH meter. Chlorophyll *a*, a measure of algal concentrations, was measured as an indicator of food resources for *G. affinis*; this food availability is important in determining the breeding period of this fish species (Pyke 2005). For this analysis, 20 mL water samples were drawn from below the water’s surface at each of the five sites and passed through a glass microfiber filter (nominal pore size 0.5 μm). Each filter was folded, wrapped in aluminum foil, and frozen at − 20 °C until analyzed. Extraction and analysis of chlorophyll *a* were carried out in low light to avoid chlorophyll degradation. Each filter was blended in 20 mL of 90% MgCO_3_-buffered acetone using an Ozito homogenizer, and the resulting slurry was placed in a centrifuge tube. Samples were refrigerated at 4° C and left to settle for anywhere between 2 and 24 h. After this, the samples were shaken and centrifuged in a Universal 320 R centrifuge at 1600 *g* at high-brake for 10 min. A Turner Designs 10-AU fluorometer was used to measure chlorophyll *a* concentration. Fluorescence of 5 mL aliquots of solution were measured in the fluorometer. In some instances of especially high chlorophyll *a* concentration, it was necessary to dilute the solution by half. To compensate for interference from chlorophyll *a* degradation products, 150 μL of 0.1 N HCl was added to samples and the fluorescence measured again.

Once fish were captured and counted, they were transported to the laboratory and anaesthetized with an overdose of benzocaine. Once the fish were deceased, they were measured for body length, weighed, their sex determined based on the presence or absence of the gonopodium, and their outer surface was inspected for parasites using an Olympus SZ40 dissecting microscope. Gills were then removed and examined under the microscope for parasites. Identification of the monogenean parasites is described in Renner and Duggan (2023).

### Statistical analyses

Prevalence and intensity of *S. seculus* were analyzed according to Bush et al. (1997), where the prevalence is the proportion of host individuals infested by a given parasite species and the intensity is the number of parasites of a single species occurring on an infested host. A binomial generalized linear mixed model (GLMM) was used to analyze the effect of the environmental factors and the fish length, and sex on the prevalence of *S. seculus*, using sampling location as a random factor. Weight was excluded from the analysis due to its close relationship with length. A time lag was applied to the temperature such that in the model for prevalence on a given month P_m_ temperature would be T_m-1_. Fish length and chlorophyll *a* concentration were log_10_ transformed to improve normality. This was repeated for the intensity of *S. seculus*, using a Poisson GLMM. All analyses were performed in R version 4.3.1 (R Core Development Team 2023). An analysis of infestation by *Gyrodactylus gambusiae* was not performed as the number observed was too low to be meaningful.

## Results

**Fig. 2:**
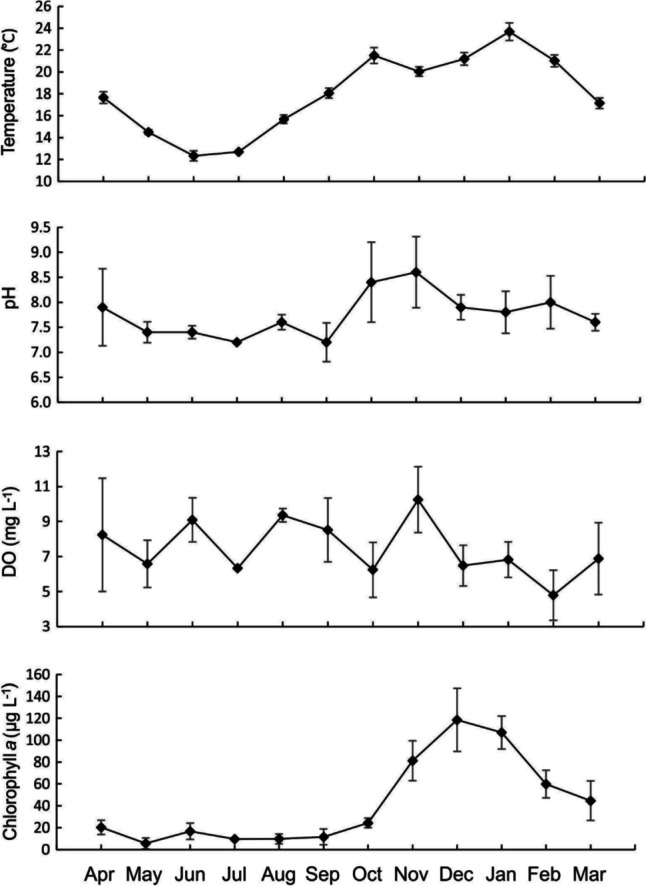
Average (+/1 SD) water temperature (°C), pH, dissolved oxygen concentration (mg L^−1^), and chlorophyll *a* concentration (μg L^−1^) across the five sampling sites from the University of Waikato, Hamilton campus lakes from April 2022 to March 2023

The lowest average water temperature of 12.3 °C was recorded in June 2022 (austral winter), rising through time gradually until reaching a maximum of 23.7 °C in January 2023 (mid-austral summer; Fig. 2). The lowest pH levels were recorded in June and September of 7.2 and was highest in November, of 8.6. The concentration of dissolved oxygen increased and decreased from month to month, with no discernible pattern. Chlorophyll *a* concentration was low from April through October, with a minimum occurring of 5.6 μg L^−1^ in May, while the maximum concentration, 118.5 μg L^−1^, was recorded in December (early summer).

**Fig. 3:**
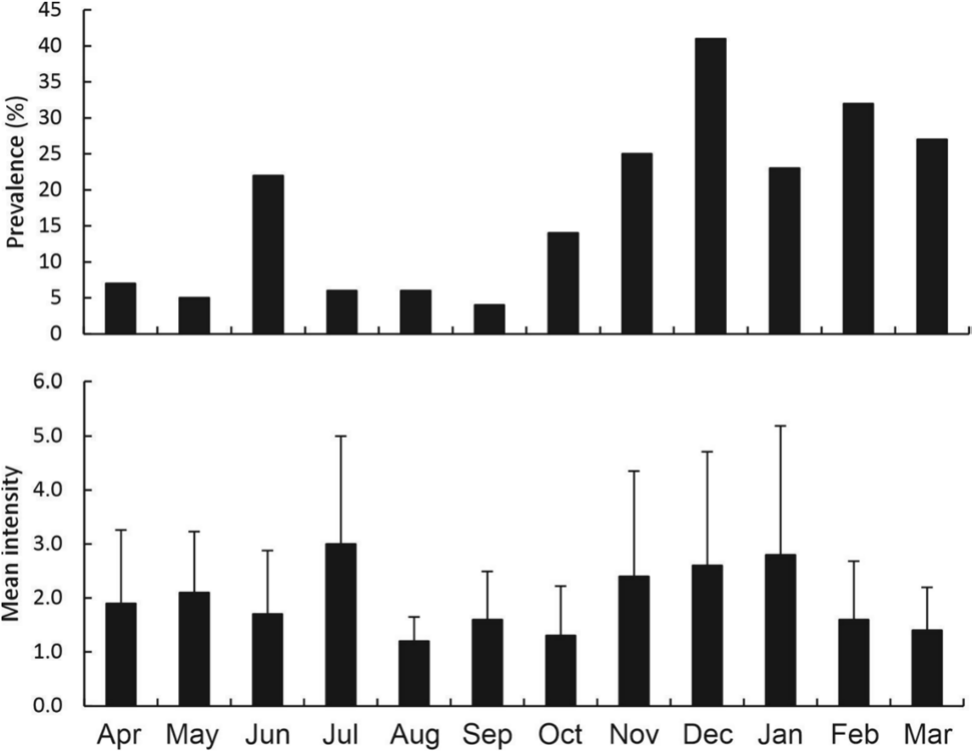
Prevalence (top) and mean intensity (bottom) of infestation of *G. affinis* by *S. seculus* from the University of Waikato, Hamilton campus lakes from 2022 to 2023

Across the course of the year, 1443 *G. affinis* were examined for monogenean parasites, and from these 551 *S. seculus* individuals and 14 *Gyrodactylus gambusiae* individuals were encountered. The highest prevalence of *G. gambusiae* occurred in March 2022, at 2.9%, and the intensity was 1.0 in all but one instance in March, when two *G. gambusiae* individuals were observed on a single fish. The prevalence of *S. seculus* changed over the course of the year, being highest in December 2022 when 41.0% of fish were infested, and lowest in September at 4.0% (Fig. 3). The mean intensity did not exhibit a clear pattern and was high in both late autumn and early to mid-summer (Fig. 3). The mean intensity was highest in July 2022 (winter), with an average of 3.0 *S. seculus* individuals per infested host, while the second highest level was recorded in December 2022 (summer), with an average of 2.6 *S. seculus* individuals per infested host. The highest intensity for any individual *Gambusia affinis* was in January, when one fish was found to host to twelve *S. seculus* individuals.

From the ponds, 503 male and 790 female *G. affinis* were examined for parasites over the course of the year. The average length of male fish was 2.5 cm and the average weight was 0.12 g. The average length of female fish was 2.9 cm and the average weight was 0.23 g.

Three variables were significant predictors of the prevalence of *S. seculus* in the GLMM: chlorophyll *a* concentration, fish length, and fish sex (Table 1). Chlorophyll *a* concentrations and length of were both strong significant positive predictors of *S. seculus* prevalence. A total of 61 *S. seculus* individuals were found infesting males and 473 were from females, with a prevalence of 9.34 and 27.31%, respectively. The GLMM indicated a strong relationship for sex, with female fish infested by *S. seculus* at a significantly greater frequency than males. No significant relationship was found between prevalence and temperature.

**Table 1:** Statistical results of the GLMM analyzing the effect of fish characteristics (length, sex) and environmental variables (temperature, pH, dissolved oxygen, chlorophyll *a*) on the prevalence and intensity of *S. seculus*

	Estimate	Std. error	*z* value	*P* value
Prevalence				
Intercept	− 7.038	1.664	− 4.228	< 0.001
Length	2.011	1.325	4.351	< 0.001
Sex (male)	− 0.806	0.205	− 3.930	< 0.001
Temperature	0.048	0.041	1.187	0.235
pH	0.172	0.250	0.687	0.492
Dissolved oxygen	− 0.043	0.048	− 0.908	0.364
Chlorophyll *a*	0.854	0.253	3.374	< 0.001
Intensity				
Intercept	− 0.454	1.0708	− 0.424	0.671
Length	2.012	0.7006	2.872	< 0.005
Sex (male)	− 0.362	0.1543	− 2.348	0.019
Temperature	− 0.068	0.0277	− 2.446	0.014
pH	0.063	0.1692	0.373	0.709
Dissolved oxygen conc.	0.001	0.0360	0.026	0.980
Chlorophyll *a*	0.635	0.1815	3.497	< 0.001

Chlorophyll *a*, temperature, fish length, and sex were all significant predictors of intensity. Of the environmental variables, chlorophyll *a* was most strongly positively associated with the intensity of infestation, while the relationship with temperature was weak, and negative. Female individuals (mean = 2.19, SD = 7.14) were infested with greater numbers of *S. seculus* than males (mean = 1.30, SD = 0.69). Fish length had a positive relationship with intensity.

## Discussion

The prevalence of *S. seculus* showed a noticeable seasonal pattern, being generally higher in late spring and summer than in autumn and winter. However, the changes in mean intensity were more erratic and was relatively low throughout the study. With occasional exceptions, infested fish generally had only 1–3 *S. seculus* individuals on their gills throughout the year.

The high prevalence of *S. seculus* in summer reflects what has been observed for monogenean taxa from temperate continental climates (Ozer and Erdem 1999; Aydogdu 2006; Poulin 2020), despite New Zealand’s narrower temperature range. That prevalence would correlate with algal concentrations is, therefore, not surprising. Nevertheless, the relationship between temperature and the prevalence of *S. seculus* was not significant, which makes this pattern more complicated. Due to seasonal changes, temperature is commonly implicated in the changes in monogenean prevalence elsewhere (Valtonen et al. 1990; Ozer and Erdem 1999; Dávidová et al. 2005; Aydogdu 2006; Blažek et al. 2008). In our study, maximum temperatures were recorded in January 2023, while the maximum prevalence of *S. seculus* was observed in December 2022. In contrast, algal concentrations increased from October to a maximum in December 2022, mirroring the pattern observed for *S. seculus* prevalence. It is reasonable to propose that this correlation is observed because the fish numbers become greater through their breeding season, which extends from mid-spring to mid-autumn (Pyke 2005). Previous work on *Carassius auratus* (L.) in closed pond systems has found that the size of the host population is an important predictor of monogenean infestation (Bagge et al. 2004). An increase in algal concentrations represents a greater food resource for grazing zooplankton, which in turn are an important dietary item for *G. affinis*; this food availability is important in determining the breeding period of this fish species (Pyke 2005). For example, under experimental conditions of increasing ration size, the somatic and gonadal growth of *G. affinis* have been observed to increase, while the time to spawning decreases (Vondracek et al. 1988; Zhu et al. 2015). *Gambusia affinis* became more visible in large shoals over the summer months until mid-autumn, likely due to this annual population increase. With the assistance of hand netting, many *G. affinis* were caught over these months, even when the traps yielded few or no fish, as many individuals were gathered under vegetation, which was possibly a diurnal change or associated with breeding (Maglio and Rosen 1969; Winkler 1979; Pyke 2005). It is not uncommon for capture rates to vary seasonally, independently of population size, due to changes in behaviors, spawning activity, food availability, and physiology across seasons (Mehdi et al. 2021). Overall, the patterns of prevalence appear to largely coincide with individuals coming into close contact during the act of courting and breeding, which will promote the transmission of parasites (Pyke 2005). While our study provides evidence of seasonal variation of monogenean prevalence in New Zealand, we only present a single year of data. A multi-year investigation is required to better elucidate this pattern. Further, temperature was measured at a single time each month for each sampling site. It is possible that with continuous temperature measurements throughout the month, the effect of temperature on monogenean infestation may have appeared different.

Of the environmental variables, chlorophyll *a* was inferred to be strong significant positive predictor of intensity of *S. seculus*, and temperature a weak negative influence. For chlorophyll *a*, high intensity measurements in November to January coincided with the highest levels of algal concentrations. For temperature, the coldest months observed were June and July (austral winter), while the mean infestation intensity in July was the highest for any month. Increases in monogenean numbers during periods of low temperature have been observed elsewhere (Chubb 1977). Nevertheless, it is difficult to speculate why low temperatures might coincide with the short but significant increase in mean intensity in July. Further, intensity was low overall throughout the study and change through time was erratic.

Female *G. affinis* had a greater prevalence and mean intensity of *S. seculus* than males, which did not support the hypothesis of lower male immunocompetence in this species. For example, Pickering and Christie (1980) observed greater prevalence and intensity of monogeneans in male brown trout (*Salmo trutta*). These species do have contrasting mating systems, however. For example, female *G. affinis* make a large investment in reproduction and are pursued aggressively by males, which may contribute to reduced immunocompetence in this case. Because the prevalence of *S. seculus* was higher over the summer breeding period, it is possible that females were preferentially infested due to a trade-off in resources. Breeding females likely invested more energy in reproduction at the cost of immunocompetence, leading to greater parasite infestation. Apart from host sex, fish length was also a major predictor of both intensity and prevalence. As such, that females were the hosts of the majority of *S. seculus* individuals observed may be related to the size discrepancy between male and female *G. affinis*, as the female fish were considerably larger (McDowall 1990; Pyke 2005). This is consistent with fish length and weight acting as positive predictors for parasitological indices elsewhere. At first glance, the size to parasite relationship in *G. affinis* seems a simple one, as larger fish present larger and higher quality habitat patches (Muir 1969; Kuris et al. 1980), and bigger targets for swimming oncomiracidia, although the relevance of fish size to parasite abundance is difficult to determine (Poulin 2000; Poulin 2013). However, there is more to be considered here; *G. affinis* have a short life cycle, typically reaching an age of 12 to 15 months, with a maximum of age of 18 months only in rare cases (Pyke 2008). Fish that have reached 50 mm in length are likely to represent particularly mature individuals (McDowall 1990), and should therefore be more immunologically competent (Izhar and Ben-Ami 2015; Izhar et al., 2020). In fact, for this reason, younger fish are expected to be parasitized more than older fish (Ashby and Bruns 2018; Wunderlich et al. 2022). It is thus difficult to be certain why the larger fish had the greatest prevalence and intensity of *S. seculus*. It is again possible that this was because many of the larger fish were reproducing females of low immunocompetence. In the case of short-lived fish like *G. affinis*, these patterns may be a result of the time required to acquire parasites. Though the young fish may be more vulnerable to infestation by monogeneans, *G. affinis* grow rapidly (Pyke 2005) and, therefore, may already be of a considerable size before monogeneans have had an opportunity to attach themselves. Alternatively, it is perhaps an effect of the current study focusing on a single parasite taxon, and should the view be broadened to the parasite communities of *G. affinis*, one might find younger fish to host more parasites overall. As weight is related to the length of the fish, it is worth noting that without examining the fish for all parasite taxa, it cannot be known for certain what portion of the mass is contributed by the fish and what portion is contributed by internal parasites (Timi and Poulin 2020). A heavier fish may already experience morbidity from an unseen parasite load that could predispose it to infestation or infection by further parasites. It is possible that such a situation contributed to the greater *S. seculus* intensity in larger *G. affinis* but the extent of such a contribution would require further research.

Of the two monogenean species observed in this study, the surface dwelling *Gyrodactylus gambusiae* was by far the least prevalent. Why this species had such a low frequency of occurrence is difficult to ascertain as there does not appear to be any reason to expect greater monogenean numbers on the gills than the skin (Scheifler et al. 2022). *Gyrodactylus gambusiae* has remained little studied since its initial description. However, Carpenter and Herrmann (2020) examined parasite communities of *Gambusia affinis* in Texas and observed *S. seculus* and an unidentified *Gyrodactlyus* species. This was likely *G. gambusiae*, however, as this is the only *Gyrodactylus* species known to infest *Gambusia affinis* (Hoffman 1999). In that instance, also, the abundance of *Gyrodactylus* was considerably lower than that of *S. seculus*. Differences in transmission may be responsible for this discrepancy. *Salsuginus seculus*, like most monogenean species, is oviparous (Whittington and Chisholm 2008), and releases eggs directly into the water column, which hatch into free-swimming larvae that then find their host. *Gyrodactlyus*, in contrast, is a viviparous genus that lacks a specific transmission stage. Instead, parents give birth to crawling, sexually mature young, that attach onto the same host as their parent (Bakke et al. 2007). Transfer from one host to another is usually not undertaken by the parasites as it is a risky maneuver that can result in high mortality (Bakke et al. 2007; Tepox-Vivar et al. 2022). However, transmission by *Gyrodactlyus* may be achieved in a number of other ways. If a host and prospective host make physical contact then monogeneans may cross from one individual to the other (Bakke et al. 1992; Bakke et al. 2007). These monogeneans may also detach and drift into the water column where they may remain as long as possible, waiting for a potential host, or they may attach themselves to a substrate to wait for a prospective host (Bakke et al. 1992; Bakke et al. 2007). These are, then, two monogenean species with distinctly different abilities to transmit from host to host. The free-swimming larval stage of *S. seculus* may have lower mortality in its transmission stage than *G. gambusiae*, though *G. gambusiae* has more strategies open to it. In other instances, *Gyrodactylus* has been observed to spread extensively through host populations (Cone and Roth 1993; Appleby 1996; Mo, 1997; Dávidová et al. 2005). The fact that *G. gambusiae* had a lower prevalence than *S. seculus* may be due to having fewer potential attempts at transmission, and *G. affinis* being a short-lived fish species with less time to accumulate parasites. Instead, *G. gambusiae* individuals might be expected to experience higher aggregation and proliferate on single hosts, leading to heavily burdened individuals with higher mortality. One heavily burdened individual of *Gambusia affinis* was observed in April 2022 (ICD, personal observation), though we did not observe this during our seasonal survey. It is also probable that some *Gyrodactylus gambusiae* will have abandoned their hosts when euthanized. Benzocaine has been found to reduce abundances of monogeneans infesting fish elsewhere (Diggles et al. 1993; Trujillo-Gonźalez et al. 2018; Vercellini et al. 2023). As such, it may be informative for future researchers to pass water in which fish are euthanized through a fine mesh to observe for detached monogeneans.

*Gambusia affinis* is a highly invasive fish and has colonized freshwaters globally (Pyke 2008). During introduction, parasite species to which it plays host may be co-introduced to new localities. The possibility that spillover of these monogeneans into populations of native New Zealand freshwater fish remains a question for future research. Monogeneans are typically considered highly host specific (Poulin 1992), with a low probability of spillover to native species (Sheath et al. 2015; Costa et al. 2018). Nevertheless, spillover to native species from invaders has been observed elsewhere. For example, using DNA barcoding, monogeneans have been observed to move hosts from non-native Nile Tilapia to native cichlids in sub-Saharan Africa (Geraerts et al. 2023). As the diversity of monogeneans infesting New Zealand native fish species are practically unknown (Renner and Duggan 2023), they may represent some risk.

To conclude, a number of factors were considered that might affect the prevalence and intensity of monogeneans on *Gambusia affinis* in relation to seasonal changes and host characteristics. Abiotic conditions did not appear to have a great effect on the populations of *S. seculus*, but seasonal variations were still observed as found in locations where temperatures vary to a greater degree. Instead, it appears that the seasonal population increase in *G. affinis* and their close association when breeding may have led to increases in the prevalence of *S. seculus*. The size of *G. affinis* was a predictor of prevalence and intensity of *S. seculus*. The possible reasons for this are diverse but the simplest explanation is that larger host individuals represent better habitat patches for ectoparasites, with larger animals having larger gills. The time available for parasites to accumulate is also greater for older fish, which have been alive longer. This research has extended the knowledge of monogeneans of *G. affinis* and represents the first study on the ecology of *S. seculus* and *G. gambusiae*. It also extends the knowledge of monogeneans in New Zealand and has utilized this environment to examine parasite seasonality in a different climate from those where most previous studies have been conducted.

## Data Availability

The experimental data are available from the authors on reasonable request.
